# Closed Genome Sequence of *Chryseobacterium piperi* Strain CTM^T^/ATCC BAA-1782, a Gram-Negative Bacterium with Clostridial Neurotoxin-Like Coding Sequences

**DOI:** 10.1128/genomeA.01296-17

**Published:** 2017-11-30

**Authors:** Travis G. Wentz, Tim Muruvanda, Sara Lomonaco, Nagarajan Thirunavukkarasu, Maria Hoffmann, Marc W. Allard, David R. Hodge, Segaran P. Pillai, Thomas S. Hammack, Eric W. Brown, Shashi K. Sharma

**Affiliations:** a Division of Microbiology, Office of Regulatory Science, Center for Food Safety and Applied Nutrition, Food and Drug Administration, College Park, Maryland, USA; b Chemical and Biological Defense Division, Department of Homeland Security, Washington, DC, USA; c Office of Laboratory Science and Safety, Food and Drug Administration, Silver Spring, Maryland, USA

## Abstract

Clostridial neurotoxins, including botulinum and tetanus neurotoxins, are among the deadliest known bacterial toxins. Until recently, the horizontal mobility of this toxin gene family appeared to be limited to the genus *Clostridium*. We report here the closed genome sequence of *Chryseobacterium piperi*, a Gram-negative bacterium containing coding sequences with homology to clostridial neurotoxin family proteins.

## GENOME ANNOUNCEMENT

Botulinum neurotoxin (BoNT), its genomic neighbor and paralog nontoxic/nonhemagglutinin (NTNH), and the related tetanus neurotoxin (TeNT) form a family of horizontally mobile, proteinaceous exotoxins produced by several species of toxigenic *Clostridia*, which act via the disruption of vesicle exocytosis in the neuronal member of neuromuscular junctions through the proteolysis of SNARE proteins, which are essential to said process (1–3). Recently, a gene cluster containing coding sequences with homology to clostridial neurotoxins (CNTs) was identified within the Gram-positive bacterium *Weissella oryzae* strain SG25, and further characterization of a construct derived from the predicted light chain indicated cleavage of rat VAMP2 *in vitro* (4–6).

We identified several putative CNT homologs within the published genome of *Chryseobacterium piperi* strain CTM^T^, a Gram-negative member of the phylum *Bacteroidetes* and originally discovered in sediment samples from a freshwater creek in Pennsylvania, USA (7). CNT-like proteins were initially identified via queries of complete and partial protein BoNT coding sequences using PSI-BLAST against the NCBI nonredundant protein database on default settings (8, 9). Domain structure, motif analysis, and multiple alignment of high-scoring matches suggested the presence of multiple putative coding sequences with homology to CNTs in discrete gene clusters across three contigs of the same organism. In the existing *C. piperi* assembly (accession number GCA_000737775), several of these gene clusters and genes occurred at the ends of contigs (7). The lack of flanking sequences limited the interpretation of both the genomic context in which the gene clusters exist and an analysis of the integrity of the coding sequences for several of the genes themselves, both critical to exploring signs of horizontal sequence transfer and investigating potential CNT homology.

We announce the closed genome sequence of *C. piperi* strain CTM^T^/ATCC BAA-1782/CFSAN055505. A freeze-dried sample of *C. piperi* was obtained from the American Type Culture Collection (ATCC, Manassas, VA) (BAA-1782) in September 2016. All DNA extraction was performed via the DNeasy blood and tissue kit (Qiagen, Inc., Valencia, CA) on overnight culture grown at 30 ± 2°C in tryptic soy broth. Initially, to verify the presence of CNT-like coding sequences (CDSs) within the *C. piperi* genome, DNA extracts were sequenced on a MiSeq platform using a 2 × 250-bp paired-end Nextera XT sample prep kit (Illumina, Inc., San Diego, CA). CLC Genomics Workbench 9.0.1 (Qiagen) was used to trim and assemble reads into contigs with ~40× mean coverage over a 4.3-Mbp genome (*N*_50_, 45,828 bp) and to conduct a local BLAST search against the putative CNT-like gene clusters. To produce the closed genome, a 10-kb genomic library was prepared, size-selected via BluePippin (Sage Science, Beverly, MA), and sequenced on an RSII sequencing platform (Pacific Biosciences, Menlo Park, CA), as per the manufacturer’s specifications. *De novo* genome assembly of sequenced reads was carried out via HGAP version 3 (Pacific Biosciences). The MiSeq short reads were utilized in conjunction with the program Pilon to improve accuracy (10). The assembly resulted in a single closed chromosome spanning 4,501,946 bp, with mean coverage of 250× and 35.3% GC content. The resulting chromosome was evaluated for methylation patterns associated with nucleotide motifs and annotated via the NCBI Prokaryotic Genome Annotation Pipeline (11).

### Accession number(s).

The complete genome sequence of *Chryseobacterium piperi* strain CTM^T^/ATCC BAA-1782/CFSAN055505 has been deposited in DDBJ/ENA/GenBank under the GenBank accession number CP023049.
